# Citizens' Options When Accessing and Sharing Health Information – An International Survey of IMIA Member Countries

**DOI:** 10.1055/s-0044-1800760

**Published:** 2025-04-08

**Authors:** Camilla Hjermitslev, Helen Monkman, Julia Adler-Milstein, Thomas Schmidt, Christian Nøhr, Jeppe Eriksen

**Affiliations:** 1 Department of Sustainability and Planning, Aalborg University, Denmark; 2 School of Health Information Science, University of Victoria, Canada; 3 Department of Medicine, University of California San Francisco, USA; 4 SDU Health Informatics and Technology, The Maersk Mc-Kinney Moller Institute, University of Southern Denmark, Odense, Denmark; 5 Department of Biomedical Informatics, Jacob's School of Medicine and Biomedical Sciences, University at Buffalo, USA

**Keywords:** Consumer Health Informatics, Patient-generated Health Data, Accessibility, Personal Health Information, International Medical Informatics Association

## Abstract

**Introduction** : Citizens' access to personal health information and information on prescription medication, options to share personal health data, and how these personal health data are kept secure, are all important themes in health informatics and therefore elaborated upon in this paper.

**Methods** : The empirical data stems from a survey that examines citizens' temporal access to laboratory test results, options for sharing patient-generated health data (PGHD) with health providers, methods to obtain supplementary information on prescription medication, and security issues pertaining to national standards, education, and experienced breaches.

**Results** : Results are based on answers from representatives in the International Medical Informatics Association (IMIA) member countries (n=28). Data shows that citizens' online access to test results is possible as soon as they are available in ten countries whereas nine countries have no norm or standard. The most common ways to provide citizens with supplementary information on prescription medication is through package inserts from manufacturers or paper medication information from pharmacies. PGHD is shared primarily in print or by showing the device to the health provider. Regarding e-health security, most countries have national standards for the security, however, less than half of the IMIA representatives answer that health professionals are required training in the national standards. Lastly, 16 of the 28 answers reply that there has been leaks leading to unauthorized access to health data. Future research should focus on how to provide citizens access to lab results according to their needs and examine how to include digital PGHD meaningfully into clinical practice.

## 1. Introduction

According to the World Health Organization's (WHO), ‘Global Strategy on Digital Health 2020-2025’, “Digital health should be an integral part of health priorities and benefit people in a way that is ethical, safe, secure, reliable, equitable and sustainable. It should be developed with principles of transparency, accessibility, scalability, replicability, interoperability, privacy, security, and confidentiality” [ 1 ].

In other words, many factors need to be considered analytically when assessing the impact of digital health and to what extent it benefits the users.

Digital technologies are believed to be essential to secure sustainable health systems, and digital health should be applied to strengthen and scale up health promotion, disease prevention, diagnosis, and more [ 1 ]. Regarding the disease prevention, precision prevention has been an emerging trend in the last decade [ 2 , 3 ]. To achieve and enhance precision prevention, digital health and health technologies are useful to obtain health information from *e.g.* , mobile health applications, digital wearables, and electronic health records [ 4 ]. However, the use of digital health data may induce privacy concerns for the citizens, thus it is important to secure, *i.e.* , trust, transparency, and robust policies, and make sure the citizens recognize the benefits of sharing health data [ 4 ].

In this paper, focus is primarily on accessibility and security, with an emphasis on how digital health needs to benefit the citizens.

Citizens' access to their own health records and their ability to contribute self-collected data to their health record, known as Patient-Generated Health Data (PGHD), is pivotal, considering how digitization shapes current healthcare systems and the emphasis on citizens self-managing their chronic conditions [ 5 6 7 ]. Although accessibility in several countries is considered a human right, research studies indicate differences in the available personal health information and methods of sharing [ 5 , 8 ].

Citizens' access to electronic health records (EHRs) including laboratory test results is a field with new regulations and continuing progress in countries around the world. However, the implementation of national regulations is not necessarily sufficient to ensure that citizens are granted full access to their health data [ 5 ]. For example, other barriers like the interoperability of the information systems and stakeholders' willingness to comply with the regulations [ 5 , 6 ] also need to be taken into account.

How citizens make use of online health information, and the potential implication of this access is another research field [ 1 , 5 , 6 ]. On the one hand, studies show that access to health records improves patients' understanding of their conditions and how they self-manage these conditions providing them with a feeling of control [ 5 , 6 ]. On the other hand, full access may increase inequity in health care due to variances in patients' abilities to use digital devices, mental status, and health literacy [ 1 , 5 , 6 ].

Other issues concern how PGHD can be shared with health providers and used in clinical practice [ 9 10 11 ]. PGHD is a broad and expanding part of health care, ranging from wearable devices, apps, and blood pressure monitors at home to questionnaires measuring patient-reported outcomes (PROs) [ 9 , 12 , 13 ]. Studies show that when using PGHD, in this case, PROs, actively in clinical practice, patients gain a positive effect on their sense of control, adherence to drug regime and an enhanced self-management; however, these effects of PROs are contextual and depend, among other things, on clinicians' and patients' knowledge, attitudes and expectations, and organizational factors [ 13 , 14 ].

As health care is becoming more digitalized and treatment strives towards patients self-managing especially their chronic conditions, PGHD can be of importance and thereby the way of sharing these data with the health provider [ 6 , 10 ]. Standards and “best practices” are needed to promote the use of PGHD in clinical practice, where technical infrastructures; inability to incorporate PGHD into clinical workflows; data privacy; doctor-patient confidentiality; and safety of mHealth apps are known barriers [ 9 , 10 , 12 13 14 ].

Security is another theme, that is focal in digital health policies, in the digital health strategy coined by WHO it is stated that “…health data are to be classified as sensitive personal data, or personally identifiable information, that require a high safety and security standard. Therefore, it stresses the need for a strong legal and regulatory base to protect privacy, confidentiality, integrity and availability of data and the processing of personal health data, and to deal with cybersecurity, trust building, accountability and governance, ethics, equity, capacity building and literacy, ensuring that good quality data are collected and subsequently shared to support planning, commissioning, and transformation of services” [ 1 ].

Considering current challenges pertaining to citizens' access to data, information acquisition, sharing of PGHD and security challenges, this study examines citizens' access to lab results and supplementary information on prescription medication, how citizens share PGHD with their healthcare providers, the existence of national standards for eHealth security, to what extent healthcare professionals (HCPs) are educated on IT-security and whether the represented countries have experienced security breaches. All these topics are examined from the perspective of International Medical Informatics Association (IMIA) representatives. The outcomes of the study are relevant input to policy makers who must ensure initiatives that cater for patients' access to their own health data as it is essential to enhance precision prevention.

## 2. Method

In January 2021 a questionnaire with 13 questions was distributed via email to 90 IMIA representatives who were given four weeks to answer. The questionnaire includes six overall themes including access to own health information in the public health care systems, access to own health information in the private health care systems, test result access for citizens, communication on prescription medication, sharing of PGHD, and lastly IT security and confidentiality of personal data. Answers to six of the questions are, together with the background question, are being published in January 2023 by Eriksen *et al.* [ 15 ]. Along with the background question, the remaining six of the 13 questions are included in this study. The questions are as follows:

- Which country do you represent? (Background question)
- When are test results made available to citizens online?
- How do citizens get supplementary information about prescription medications?
- How can citizens share data from a personal electronic monitoring device or wearable device with their health provider?
- Are there national standards for how health care institutions secure and protect health data?
- Are the professionals dealing with health data in your country required to be trained in national standards for protecting health data?
- Has there been any data breach in institutions that have led to unauthorized access to health data?

To enable analytical comparisons between the regions The IMIA member countries were divided into world regions based on WHOs regional classification [ 16 ] (see Table 1 ). This division was chosen in favor of the IMIA regions to allow for future comparative studies that might include other variables.

**Table 1.** Regional distribution of answers by country and IMIA member countries.

Region	Distribution of answers by country	Relative frequency (%)	Distribution of all IMIA countries	Relative frequency (%)
Europe	12	43	31	34
Americas	5	18	10	11
Africa	4	14	18	20
Western Pacific	4	14	9	10
South-East Asia	2	7	6	7
Eastern Mediterranean	1	4	16	18
Total	28	100	90	100

## 3. Results

**Figure 1.**
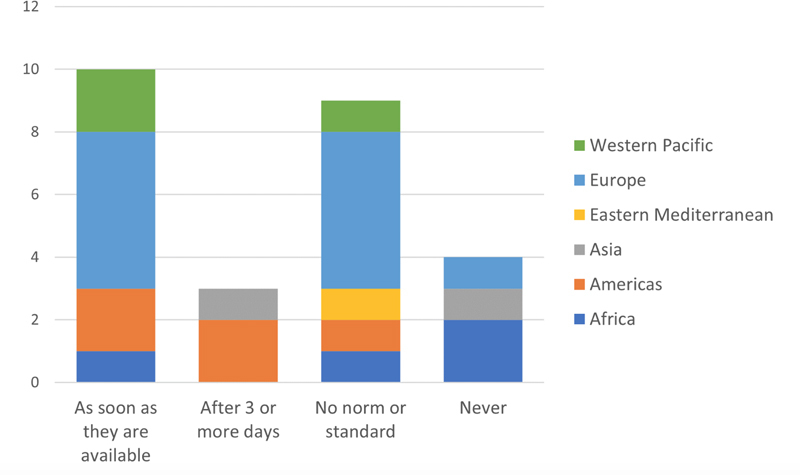
Responses about when test results are made available to citizens online (n=26).

**Figure 2.**
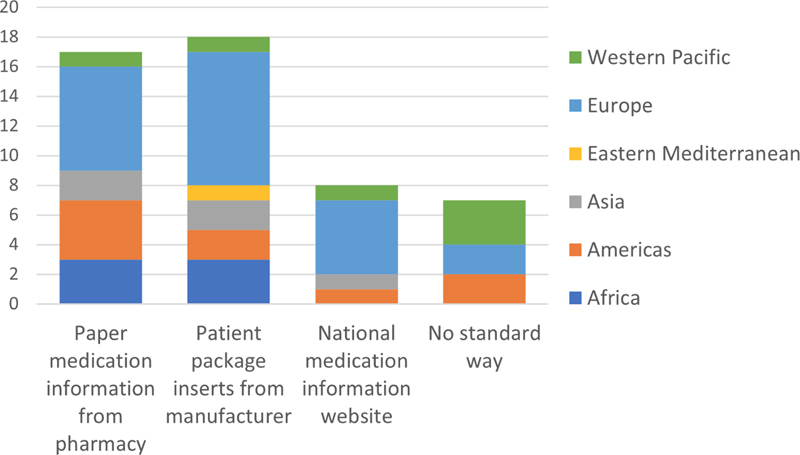
Responses about how citizens can gain more information about prescription medication (n=28)

**Figure 3.**
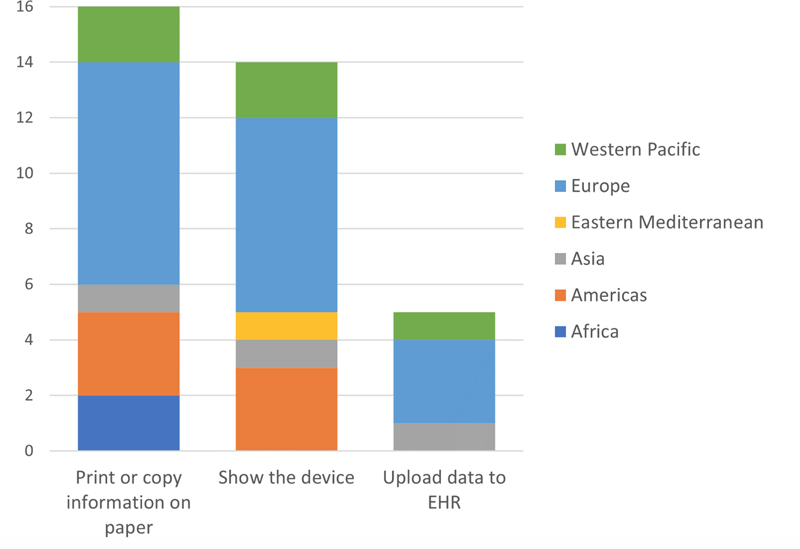
Responses how citizens can share data from personal electronic monitoring devices or wearable devices with their health provider (n=22).

**Figure 4.**
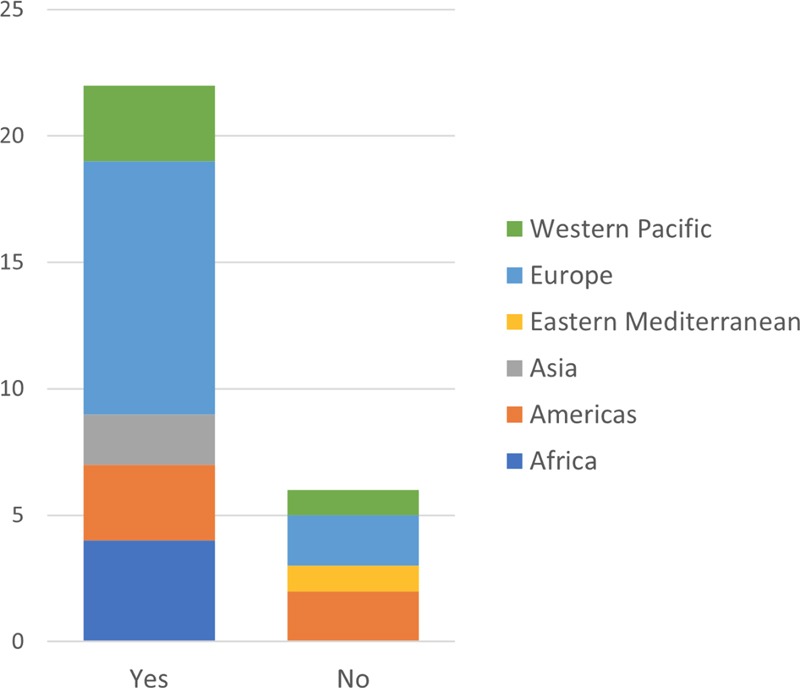
Responses regarding national standards for health data security (n=28).

**Figure 5.**
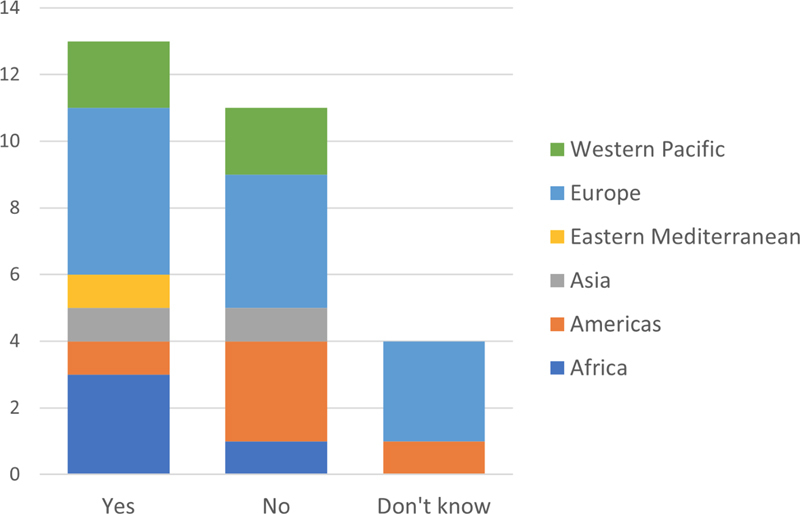
Responses whether the health professionals are required to be trained in national standards for protecting health data (n=28).

**Figure 6.**
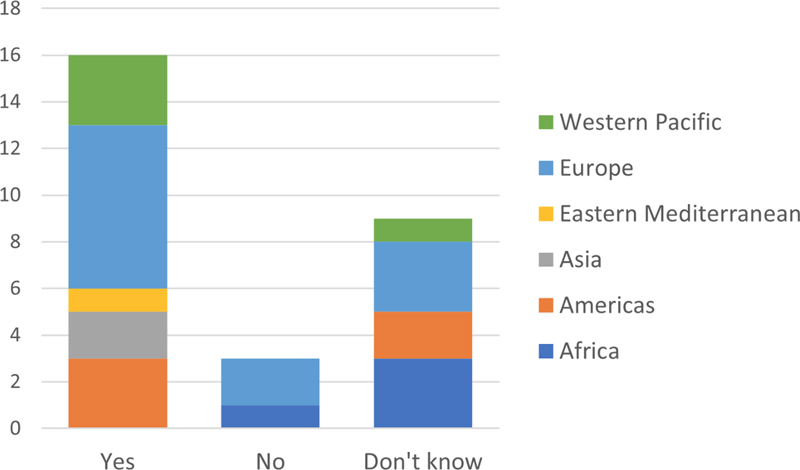
Responses on any data leaks leading to unauthorized access to health data (n=28).

IMIA representatives from 28 countries answered the questionnaire, which equals a response rate of 31%. In this section, results concerning access to test results ( Figure 1 ), access to supplementary information on prescription medication ( Figure 2 ), sharing of PGHD ( Figure 3 ), and security of personal digital health data (Figures 4-6) are presented while distributed into regions.

### 3.1. Time to Test Results Access

The IMIA representatives were asked when test results are made available for the citizens online. The two most common answers to when test results are made available to citizens online were *as soon as they are available* or *no norm or standard* (see Figure 1 ). Data show that in 10 countries in total, from Europe (5), Western Pacific (2), Americas (2) and Africa (1), citizens have access to their test results *as soon as they are available* , which is the most common practice (38%) across the participating countries. In nine countries (35%), located in Europe (5), Africa (1), Americas (1), Eastern Mediterranean (1), and Western Pacific (1), *no norm or standard* exists. In three countries (12%), from Americas (2) and South-East Asia (1), citizens can access their test results *after 3 or more days* , whereas test results *never* are accessible in four countries (15%), located in Africa (2), South-East Asia (1) and Europe (1).

### 3.2. Supplementary Information on Prescription Medications

The representatives were asked how citizens get supplementary information about their prescription medications. Answers were not mutually exclusive. That is, respondents could select multiple information sources. As seen in Figure 2 from the 28 answers, 17 of the countries provide *Paper medication information from pharmacy* , containing 75% of the African countries (3), 25% of the Western Pacific countries (1), 58% of the European countries (7), 80% of the American countries (4), and 100% of the Asian countries (2). *Patient package inserts from manufacturer* are found to be the case in 18 of the 28 countries, which includes 25% of the Western Pacific countries (1), 40% of the American countries (2), 75% of the European (9) and the African (3) countries, and 100% of the Asian (2) and Eastern Mediterranean (1) countries. Eight countries have a national medication information website, 5 countries in Europe and 1 country in Asia, Americas, and Western Pacific. Seven of the countries asked, had *No standard way* .

### 3.3. Sharing of PGHD

To investigate the possibility in different countries of sharing PGHD, the representatives were asked how citizens can share data from a personal electronic monitoring device or wearable device with their health provider. In the majority of the countries, citizens can share PGHD with their health provider by *print or copy information on paper* [ 16 ], the answer is closely followed by *show the device* to the health provider [ 14 ] (see Figure 3 ). Answers were not mutually exclusive, as such respondents could select more than one answer.

In 16 countries (67%), from Europe (8), Americas (3), Africa (2), Western Pacific (2), and South-East Asia (1), citizens can share PGHD via *print or copy information on paper* . Furthermore, data shows that in 14 countries (58%), from Europe (7), Americas (3), Western Pacific (2), South-East Asia (1), and Eastern Mediterranean (1), citizens can share PGHD by showing the device. Lastly, citizens are able to *upload data to EHR* , in five countries (21%), from Europe (3), South-East Asia (1), and Western Pacific (1).

### 3.4. National Standards for E-Health Security

IT security regarding health data is of interest with the continuous increase in using e-health. Consequently, the IMIA representatives were asked whether their countries have any national security standards for how health care institutions secure and protect health data. The majority of the respondents (79%) answer that there are national standards for e-health security in their countries. 22 of the 28 countries answer *Yes* , while the remaining six countries answer *no* , see Figure 4 . Two countries from both Europe and the Americas and 1 country from both Eastern Mediterranean and Western Pacific have no national standard.

### 3.5. IT-Security training of Health Professionals

Regarding the question whether professionals are required to be trained in national standards for protecting health data the answers are almost equally distributed between *yes* and *no* . Figure 5 shows the answers from the 28 countries of which 11 countries (39%), from Europe (4), the Americas (3), Western Pacific (2), Asia (1), and Africa (1), have no required training of health professionals for protection of health data. 13 countries (46%), from Europe (5), Africa (3), Western Pacific (2), the Americas (1), Asia (1), and Eastern Mediterranean (1), have required training of health professionals, while 4 countries from Europe (3) and the Americas (1) do not know.

### 3.6. Health Data Breaches

The IMIA representatives were asked if any data breaches have led to unauthorized access to health data and, as seen in Figure 6 , 16 countries (57%) answer they have had a data breach leading to unauthorized access, which include countries from Europe (7), Western Pacific (3), the Americas (3), Asia (2), and Eastern Mediterranean (1). Furthermore, only 3 countries (11%), from Europe (2) and Africa (1), answer they have had no breach, while 9 countries (32%) from Europe (3), Africa (3), the Americas (2), and Western Pacific (1), do not know whether a data breach has occurred leading to unauthorized access to health data.

## 4. Discussion

According to previous studies, differences in available personal health information between and within countries is a known issue [ 5 , 8 ]; variations, that are confirmed in this survey study on national and regional levels.

This lack of standardization is also reflected in the answers pertaining to citizens' online access to test results ( Figure 1 ). Notably, 50% of the countries have either *no norm or standard* or *never* provide patients with access to test results. Hence, it seems that citizens in some cases are not provided with adequate access to the test results and that some countries do not adhere to the normative standards within the field of health informatics [ 17 ].

Interestingly, standardization was less inconsistent when examining supplementary information about prescription medications ( Figure 2 ) but perhaps because analogue practices were predominant in this context. Specifically, the two most prevalent ways of communicating information about prescription medications were printed pharmacy leaflets and paper package inserts. However, eight mostly European countries had national prescription medication websites. These differences align with previously examined differences in how supplementary information about prescription medication information is offered to citizens internationally ( *e.g.* , Monkman *et al.* , 2017 [ 18 ]). A single online repository of information about prescription medication for citizens is appealing for its potential to offer citizens consistent, trustworthy, information that can be accessed from anywhere [ 19 ]. However, reported reading rates of paper leaflets are low [ 20 ] and introducing friction such as requiring citizens to seek out this information of their own volition ( *i.e.* , pull communication) may result in even fewer people reading them. Supporting this argument, one study indicated people would prefer to receive information about their prescription medications by email, sent directly to them ( *i.e.* , push communication) [ 21 ]. Given the importance of supplementary information about prescription medications, it is worthwhile exploring how best to share this information with citizens, to increase the likelihood it is read and so it offers them the most value [ 22 ].

Regarding new opportunities in consumer health informatics related to the possibility of sharing PGHD with healthcare providers ( Figure 3 ), it is remarkable how analogue options dominate considering the digitization of healthcare [ 5 6 7 ]. This finding seems to confirm continuous barriers when uploading PGHD to EHRs, underscoring the relevance of studies on interoperability between systems [ 9 , 10 ]. Especially, when considering how PGHD might benefit the patients [ 9 , 10 , 13 , 14 ]. Besides the technical requirements other factors might also explain the lacking use of PGHD in clinical practice. Taking PROs as an example, adequate knowledge and information; physicians and patients attitudes and expectations and organizational factors explain why, how and whether PRO data is used in clinical practice. If the impact of these factors is not considered, it might result in non-use or incorrect use of PROs in clinical practice. Therefore, if PROs, and probably other PGHD, are to be used routinely as part of clinical practice, education of clinicians and patients on how to interpret and use data; organizational adjustments adapting workflows and allocation of resources; technical structures enabling the use of PGHD; and transparency regarding the purpose and application of these type of data are recommended [ 13 ]. The importance of PGHD in current healthcare is underscored by the construction of European Health Data Space (EHDS) lead by the European Union (EU). The EHDS is an attempt to facilitate the access to and sharing of health data across countries and stakeholders in EU, with the aim to establish a single market in the area of digital health, allow the use of better health data in research and policy-making and empower citizens [ 23 ]. However, a concern in this context, is citizens' willingness to share their health data, which is focal as the value and functionality of PGHD is determined by patients engagement. Hence, it is interesting that a recent study conducted across the Nordic countries shows that the majority of the populations in Denmark, Sweden, Norway, Finland and Iceland are willing to share data on medical history, diagnoses, prescriptions, test results, immunization and allergies, in cases where the information is needed by healthcare providers and also for research purposes [ 24 ]. Hence, it is noteworthy, how the study in this paper shows that only five countries allow patients to upload their data. This implies an extensive gap between the current situation and the desired plateau where PGHD can be a natural part of clinical care, leading to patients being more independent and engaged in decision-making and treatments and prevention can be more personalized [ 4 , 9 , 13 , 25 ].

The responses pertaining to national standards for health data security in Figure 4 , illustrate the growing global legislative focus on ensuring the confidentiality and integrity of patient data. Although there is a natural diversity between regional and national regulatory approaches, several of the responding regions are establishing cross-border partnerships and collaborations for sharing and protecting health data infrastructure. Securing infrastructure is a key, but also costly, concern tapping often limited resources for preventive measures, governance, and legislative enforcement. A natural consequence of an interconnected digital world is legislative inspiration and cross fertilization as seen in the international emergence of data protection acts equipping citizens with rights and autonomy, data breach notification requirements, and constraints pertaining to data management.

Figure 5 , which report the extent to which respondents state that health care professionals are required to be trained in national standards for protecting health data, indicate that health care institutions in general face an ongoing challenge to ensure that employees comprehend and adhere to current laws and regulations. This challenge is a part of the ongoing transformation that digitalization and digitization impose on healthcare services and point to the necessity of embedding information security awareness into daily practices of clinicians. This lack of awareness shows from Figure 6 's percentage of responses unaware of any breaches leading to unauthorized data access. Even though the survey did not quantify any number of individuals affected by an incident before classifying it as a breach, the responses tell us that institutions face a substantial task in communicating the prevalence and risk of data breaches. Several information security awareness studies and frameworks have been published and can easily be implemented by healthcare organizations to gauge the knowledge, awareness, and behavior of their employees [ 26 , 27 ].

Though this survey study includes no more than one-third of the IMIA member countries, the results indicate that further efforts are necessary to increase citizen access to lab results and to broaden citizens' options when sharing PGHD; especially, the improvement of digital alternatives is required.

One way to overcome some of the barriers concerning citizen access to lab results and the sharing of PGHD in clinical practice might be to consult stakeholders, citizens, clinicians, developers, etc. [ 28 ]. This can be done through the application of participatory design methods, which allow the creation of designs that are based on the preferences and needs of the users [ 28 ]. Hence, we encourage the application of participatory design methods in future studies to improve citizens' accessibility to lab results and the sharing of PGHD in clinical practice. Furthermore, there is a continued need for regulations on data security and training in these for health care personnel, along with awareness of breach risks.

This survey study shows variations across countries; however, regional differences within the countries are not visible based on these data, which is a limitation. Another limitation concerns the response rate; hence, it would be relevant to conduct an additional analysis based on a larger number of answers collected from IMIA representatives.

Despite the limitations of this cross-section study, it represents a first attempt to provide insight in how different countries are dealing with the many new opportunities made available through digitalization of health data, and the rapidly growing infrastructure. The new fast and relatively standardized infrastructure make access to a very large amount of data easier for clinicians, administrators, and citizens/patients. Comparative studies can provide useful information to policy makers; however, a more comprehensive use of cross section studies will be obtained when they are repeated at fixed intervals *i.e.* , monitoring dashboards.

## References

[1] WHO. Global strategy on digital health 2020-2025. World Health Organization; 2021.

[2] McGrath C, Palmarella G, Solomon S, Dupuis R. Precision Prevention and Public Health. Center for Public Health Initiatives, Data Brief. 2017.

[3] Winston F, Puzino K, Romer D. Precision prevention: time to move beyond universal interventions. Inj Prev. 2016 Apr; 22. 10.1136/injuryprev-2015-041691 26271260

[4] Canfell O, Littlewood R, Burton-Jones A, Sullivan C. Digital health and precision prevention: shifting from disease-centred care to consumer-centred health. Aust Health Rev. 2022 Jun; https://doi.org/10.1071/AH21063 10.1071/AH21063 34882538

[5] Hägglund M, DesRoches C, Petersen C, Scandurra I. Patients' access to Health Records. BMJ. 2019 Oct; https://doi.org/10.1136/bmj.l5725 10.1136/bmj.l5725 31578180

[6] Richards T, Coulter A, McMillan B, Hagglund M. Patient access to full General Practice Health Records. BMJ. 2022 Dec; https://doi.org/10.1136/bmj.o3019 10.1136/bmj.o3019 36535705

[7] Eriksen J. Purpose, Functionality and Reconceptualisation of Patient-Reported Outcome: a patient participation perspective on pro in clinical practice post its digitalisation. Aalborg Universitetsforlag. ; 2021.

[8] Essén A, Scandurra I, Gerrits R, Humphrey G, Johansen M, Kierkegaard P. Patient access to electronic health records: Differences across ten countries. Health Policy and Technology. 2017;44–56. https://doi.org/10.1016/j.hlpt.2017.11.003

[9] Omoloja A, Vundavalli S. Patient generated health data: Benefits and challenges. Current Problems in Pediatric and Adolescent Health Care. 2021 Nov; https://doi.org/10.1016/j.cppeds.2021 10.1016/j.cppeds.2021.101103 34799255

[10] Hussein R, Crutzen R, Gutenberg J, Kulnik S, Sareban M, Niebauer J. Patient-generated health data (PGHD) interoperability: An integrative perspective. Studies in Health Technology and Informatics. 2021 May; https://doi.org/10.3233/SHTI210154 10.3233/SHTI210154 34042739

[11] Adler-Milstein J, Nong P. Early experiences with patient generated health data: health system and patient perspectives. JAMIA. 2019 Oct; https://doi.org/10.1093/jamia/ocz045 10.1093/jamia/ocz045 PMC7647206 31329886

[12] Lavallee D, Lee J, Austin E, Bloch R, Lawrence S, McCall D. MHealth and patient generated health data: Stakeholder perspectives on opportunities and barriers for Transforming Healthcare. mHealth. 2020 Jan; https://doi.org/10.21037/mhealth.2019.09.17 10.21037/mhealth.2019.09.17 PMC7063266 32190619

[13] Eriksen J, Bygholm A, Bertelsen P. The association between patient-reported outcomes (pros) and patient participation in chronic care: A scoping review. Patient Education and Counseling. 2022 Jul; https://doi.org/10.1016/j.pec.2022.01.008 10.1016/j.pec.2022.01.008 35090802

[14] Gensheimer S, Wu A, Snyder C. Oh, the places we'll go: Patient-reported outcomes and Electronic Health Records. The Patient - Patient-Centered Outcomes Research. 2018 Dec; https://doi.org/10.1007/s40271-018-0321-9 10.1007/s40271-018-0321-9 29968179

[15] Eriksen J, Monkman H, Adler-Milstein J, Eriksen K, Nøhr C. Citizens' Access to Online Health Information – An International Survey of IMIA Member Countries. Medinfo Proceeding. 2024 Jan. 10.3233/SHTI231174 38270024

[16] WHO. who.int. [Online].; 2023 [cited 2024 01. Available from: https://www.who.int/countries/

[17] IMIA. The IMIA Code of Ethics for Health Information Professionals. [Online].; 2016. Available from: https://imia-medinfo.org/wp/imia-code-of-ethics/

[18] Monkman H, Nøhr C, Kushniruk A. A Comparison of Danish and Canadian Consumer Medication Information. Studies in Health Technology and Informatics. 2017;: 147-152. 28809198

[19] Pearsall BM,AR,HD. Essential Medication Information for Patients: Ensuring Access. Therapeutic Innovation & Regulatory Science. 2014. 10.1177/2168479013507437 30227511

[20] Hammar T, Nilsson A, Hovstadius B. Patients' views on electronic patient information leaflets. Pharm Pract (Granada). 2016 Jun; https://doi.org/10.18549/PharmPract.2016.02.702 10.18549/PharmPract.2016.02.702 PMC4930857 27382423

[21] Monkman H, Kushniruk A, Borycki E, Sheets D, Barnett J, Nøhr C. The Medium Is the Message: How Do Canadian University Students Want Digital Medication Information? Life. 2020 Dec; https://doi.org/10.3390/life10120339 10.3390/life10120339 PMC7764253 33321799

[22] Monkman H, Kushniruk A, Borycki E, Sheets D, Barnett J. Differences in Memory, Perceptions, and Preferences of Multimedia Consumer Medication Information: Experimental Performance and Self-Report Study. JMIR Human Factors. 2020 Dec; https://doi.org/10.2196/15913 10.2196/15913 PMC7738255 33258780

[23] European Commission. Communication from the Commission - A European Health Data Space: harnessing the power of health data for people, patients and innovation. Strasbourg:; 2022.

[24] Eriksen J, Hjermitslev C, Tuulikki V, Harðardóttir G, Koch S, Faxvaag A, et al. A Nordic survey to monitor citizens use and experience with eHealth. Copenhagen:; 2023.

[25] Austin E, Lee J, Amtmann D, Bloch R, Lawrence S, McCall D. Use of patient-generated health data across healthcare settings: Implications for health systems. JAMIA Open. 2019 Nov; https://doi.org/10.1093/jamiaopen/ooz065 10.1093/jamiaopen/ooz065 PMC7309248 32607489

[26] Parsons K, Calic D, Pattinson M, Butavicius M, McCormac A, Zwaans T. The Human Aspects of Information Security Questionnaire (HAIS-Q): Two further validation studies. Computers & Security. 2017; 66:40-51. https://doi.org/10.1016/j.cose.2017.01.004

[27] Schmidt T, Nøhr C, Koppel R. A Simple Assessment of Information Security Awareness in Hospital Staff Across Five Danish Regions. Public Health and Informatics, Studies in Health Technology and Informatics. 2021 May. https://doi.org/10.3233/SHTI210248 10.3233/SHTI210248 34042653

[28] Kushniruk A, Nøhr C. Participatory design, user involvement and health IT evaluation. In Rigby M AE. Evidence-Based Health Informatics: Promoting Safety and Efficiency through Scientific Methods and Ethical Policy. IOS Press; 2016. p. 139-151. 27198099

